# Supplementary figures and images for: The clinical value of peripheral immune cell counts in pancreatic cancer
Source: PLoS One. 2020 Jun 15;15(6):e0232043. doi: 10.1371/journal.pone.0232043 (PMC7295193; doi:10.1371/journal.pone.0232043)

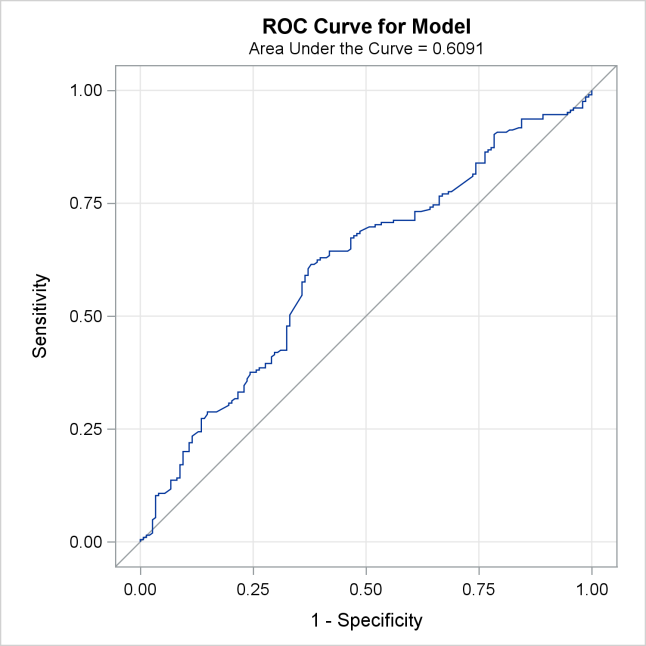

Supplement: S1 Fig — (TIF) [file pone.0232043.s002.tif]

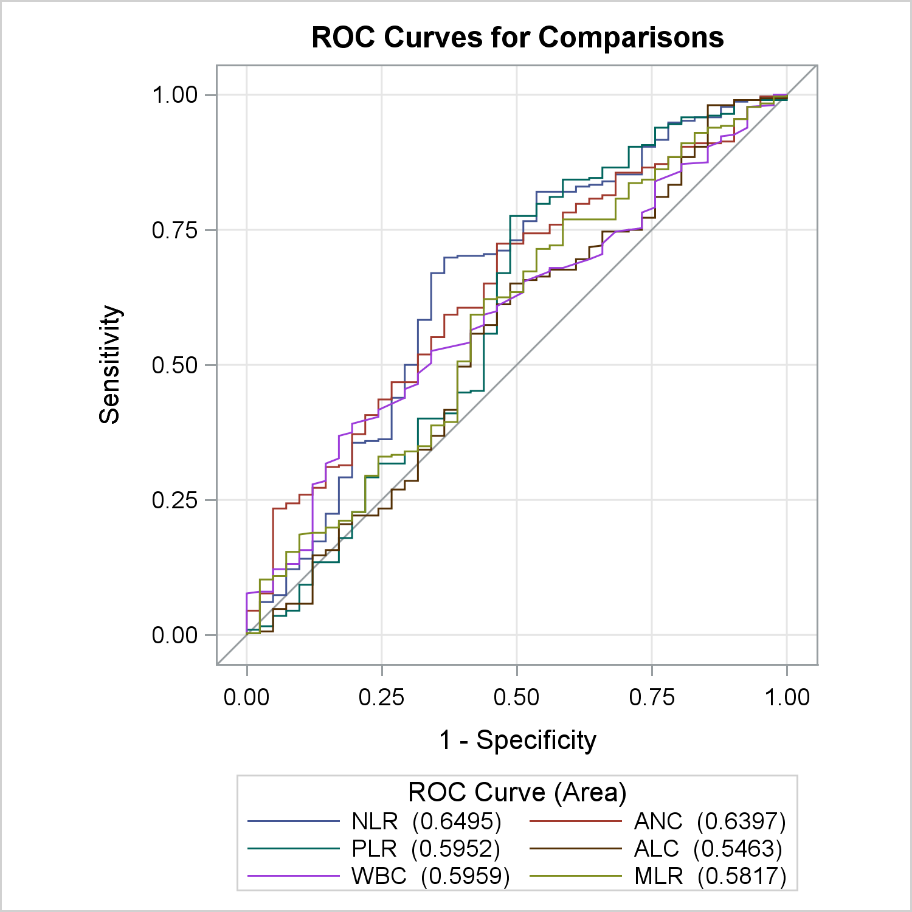

Supplement: S2 Fig — (TIF) [file pone.0232043.s003.tif]
